# Highlighted role of VEGFA in follow up of celiac disease 

**Published:** 2019

**Authors:** Sina Rezaei-Tavirani, Mohammad Rostami-Nejad, Fatemeh Montazar

**Affiliations:** 1 *Proteomics Research Center, Faculty of Paramedical Sciences, Shahid Beheshti University of Medical Sciences, Tehran, Iran*; 2 *Gastroenterology and Liver Diseases Research Center, Research Institute for Gastroenterology and Liver Diseases, Shahid Beheshti University of Medical Sciences, Tehran, Iran*; 3 *Firoozabadi Clinical Research Development Unit (FACRDU), Iran University of Medical Sciences, Tehran, Iran*

**Keywords:** Celiac disease, Biomarker, VEGFA, Follow up, Gene

## Abstract

**Aim:**

Evolution of gene expression change of intestine tissue in celiac patients to find a new molecular prospective of disease is the aim of this study.

**Background:**

Celiac disease (CD) as an autoimmune disease is known as an immune reaction response to the gluten in patients. It is reported that genetic and environmental conditions are important in onset and progress of CD.

**Methods:**

gene expression profiles of intestinal tissue in 12 celiac patients and 12 healthy controls from gene expression omnibus (GEO) were downloaded and verified by boxplot analysis. The significant and selected differentially expressed genes (DEGs) were included protein-protein interaction (PPI) network analysis. The central nodes were identified by network analyzer.

**Results:**

The network was constructed from 161 query DEGs and 50 additional neighbors. GTF2H1, VEGFA, SUMO1, RAD51, MED21, BBP4, LEP, and MAP2K7 as potent hub nodes LRP5, RABGEF1, BCAS2, DYRK1B, AOC3, RABL2A, CRTAP, VEGFA, and SPOPL as potent bottlenecks are introduced as crucial nodes.

**Conclusion:**

Among the crucial DEGs, Vascular endothelial growth factor A (VEGFA) was highlighted as an important biomarker candidate for follow up of celiac patients.

## Introduction

 One of the autoimmune diseases which is characterized by sensing and immune reaction response to gluten is celiac disease (CD) (1,2). The role of environmental and genetic factors in CD onset and promotion is confirmed and discussed in details (3). Nutrition deficiency in CD patients may lead to some disorders such as iron deficiency anemia and osteoporosis which are suffering conditions for the patients (4). 0.5%-1% of general population experience CD (5). Two diagnostic methods including initial serological screening and biopsy of small intestine are established to detect CD (3). The well-known treatment for CD is gluten free nutrition (6). Several molecular investigations about CD mechanism, diagnoses of celiac and its treatment are reported; however, there are many unknown relationships between molecular aspects (7). Recently network analysis has attracted attention of researchers. In this approach the diseases related genes, proteins, metabolites, and the other gene products are interacted as intractome units (8). Interacted network can be categorized as non-scale free and scale free networks. In the scale free networks, there are several critical nodes which can be considered as the crucial ones that are correlated to diseases (9). This method requires centrality analysis of the constructed network. Many diseases are evaluated via network analysis. Different kinds of cancers such as breast, lung, brain, and kidney tumors are investigated via PPI network analysis to find efficient diagnostic therapeutic biomarkers (10). There are several documents that are related to the analysis of diseases staging via this approach (11, 12). In the present study gene expression change of celiac patients relative to the healthy control is investigated by PPI network analysis to revile more molecular aspect of this disease. 

## Methods

Gene expression profiles of celiac patients and controls which are recorded in GEO under name of GSE112102/GPL10558 were extracted. The samples were obtained from upper GI by endoscopy examination via multiple mucosal. Intestinal multiple mucosal biopsies of 7 males and 5 females aged 18-40 years, plus 10 males and 2 females aged 21-42 years were selected as patients and controls, respectively. Gene expression distributions were investigated via boxplot analysis by GEO2R. Numbers of top 250 DEGs were identified for more analysis. FC less than 1.5, P-value more than 0.05, and uncharacterized DEGs were excluded. The remained DEGs plus 50 neighbors were included in the PPI network by Cytoscape v 3.6.0 software (13). The main connected component was analyzed based on centrality parameters. Top 10 nodes based on D, BC, CC, and S were determined to find the central genes. The hub nodes that were included in at least another group were identified as central nodes. The bottleneck nodes which were common with the other group were highlighted as crucial genes. Finally, the central and crucial nodes were evaluated to find action relationship between them. 

## Results

**Figure 1:**
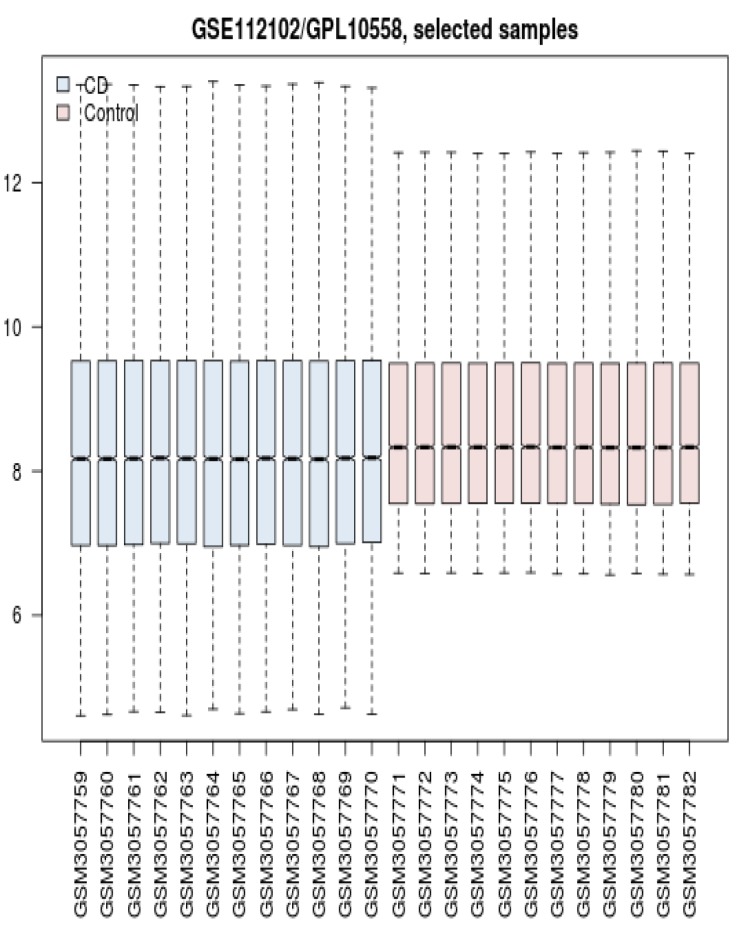
Boxplot analysis of CD patients and controls

Gene expression profiles of 12 control and 12 celiac patients were matched via boxplot analysis. As it is shown in figure 1 the profiles are comparable because middle of the samples are matched. However, distributions of patients’ expression values are more extended relative to the control samples. 

Top 250 DEGs which differentiate patients from controls were extracted and based on LogFC more than 0.6 and less than -0.6 and also p-value less than 0.05 were screened. Among 250 DEGs 170 ones were characterized and considered to import in STRING database via Cytoscape software. Numbers of 161 DEGs were recognized by STRING database that were included in PPI network. 

**Figure 2:**
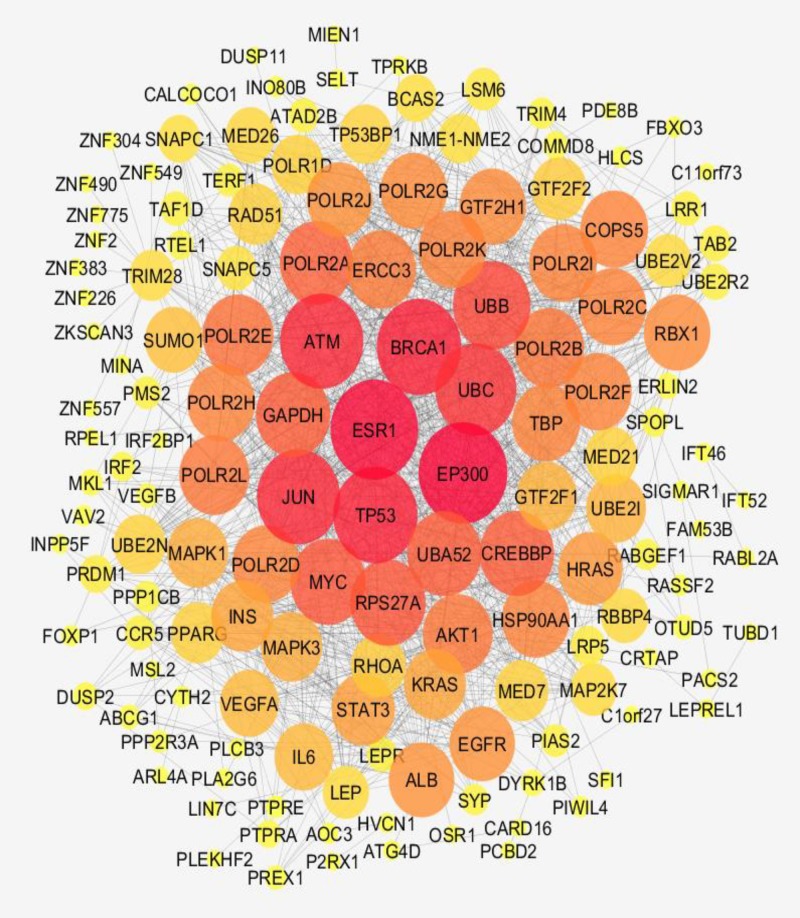
The main connected component of PPI network including 148 nodes and 1325 edges. The nodes are layout based on degree value

**Table 1:** Different networks and their details

R	Network	No. of components	No. of isolated nodes	No. of nodes in other component	No. of nodes of the main connected component	No. of query nodes included in main connected component
1	including 161 query genes	14	87	33	41	41
2	including 161 query genes + 50 neighbor genes	4	56	7	148	98
3	Including 161 query genes + 100 neighbor genes	3	46	4	211	111

**Table 2:** Hubs that are common with the top nodes based on CC. VEGFA as a hub node is presented in the groups that are selected based on BC, CC, S

R	DN	Description	D	BC	CC	S
1	GTF2H1	General transcription factor IIH, polypeptide 1, Component of the core-TFIIH basal transcription factor involved in nucleotide excision repair (NER) of DNA	38	0.007	0.468	2706
2	VEGFA^*^	Vascular endothelial growth factor A; Growth factor active in angiogenesis, vasculogenesis and endothelial cell growth.	31	0.025	0.490	7616
3	SUMO1	Ubiquitin-homology domain protein PIC1, it is involved in a number of cellular processes such as nuclear transport, DNA replication and repair, mitosis and signal transduction.	30	0.009	0.467	2936
4	RAD51	DNA repair protein RAD51 homolog 1; Fanconi anemia complementation groups	27	0.004	0.471	2290
5	MED21	Mediator of RNA polymerase II transcription subunit 21; Component of the Mediator complex, a coactivator involved in the regulated transcription of nearly all RNA polymerase II-dependent genes.	26	0.001	0.438	472
6	RBBP4	Nucleosome-remodeling factor subunit RBAP48; Core histone-binding subunit that may target chromatin assembly factors, chromatin remodeling factors.	23	0.004	0.444	1438
7	LEP	Obesity factor; Key player in the regulation of energy balance and body weight control.	22	0.004	0.461	1322
8	MAP2K7	Dual specificity mitogen-activated protein kinase 7; Dual specificity protein kinase which acts as an essential component of the MAP kinase signal transduction pathway.	22	0.001	0.457	466

**Table 3:** Bottlenecks nodes, which are common top nodes, based on stress. LRP5 is common in BC, CC, S groups. VEGFA is common between all groups

R	DN	description	D	BC	CC	S
1	LRP5	Low density lipoprotein receptor-related protein 5; Component of the Wnt-Fzd-LRP5-LRP6 complex that triggers beta-catenin signaling through inducing aggregation of receptor- ligand complexes into ribosome-sized signalsomes	14	0.041	0.440	9482
2	RABGEF1	RAB guanine nucleotide exchange factor 1; VPS9 domain containing	9	0.040	0.397	15210
3	BCAS2	DNA amplified in mammary carcinoma 1 protein; Component of the PRP19-CDC5L complex that forms an integral part of the spliceosome.	19	0.028	0.426	7546
4	DYRK1B	Dual-specificity tyrosine-(Y)-phosphorylation regulated kinase 1B; Dual-specificity kinase which possesses both serine/threonine and tyrosine kinase activities. Inhibits epithelial cell migration.	6	0.027	0.364	8882
5	AOC3	Semicarbazide-sensitive amine oxidase; Cell adhesion protein that participates in lymphocyte extravasation and recirculation.	3	0.027	0.339	7084
6	RABL2A	RAB, member of RAS oncogene family-like 2A; Plays an essential role in male fertility.	2	0.027	0.287	10032
7	CRTAP	Cartilage associated protein; Necessary for efficient 3-hydroxylation of fibrillar collagen prolyl residues; Belongs to the leprecan family.	2	0.027	0.309	6020
8	VEGFA	Vascular endothelial growth factor A; Growth factor active in angiogenesis, vasculogenesis and endothelial cell growth.	31	0.025	0.490	7616
9	SPOPL	Speckle-type POZ protein-like; Component of a cullin-RING-based BCR (BTB-CUL3-RBX1) E3 ubiquitin-protein ligase complex that mediates the ubiquitination and subsequent proteasomal degradation of target proteins.	8	0.014	0.379	6152

**Figure 3:**
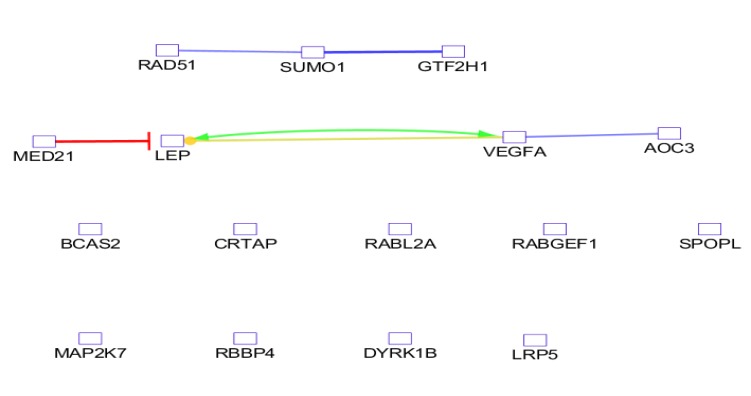
The action map for the central and crucial genes is

As it is shown in table 1 there are poor interactions between the nodes, and most of the nodes were isolated; therefore, 50 relevant genes were added and the network was constructed. Importing the 50 neighbor genes to the query genes improved interactions between the nodes, but several nodes were isolated. For better advantage, 100 relevant genes were added the query genes but result was not significantly changed. Thus, 50 additional neighbors were considered to construct the final PPI network. The network includes 4 connected components and 56 isolated nodes. Numbers of 98 query DEGs and 50 additional genes were consisted in the main connected component which are connected by 1325 edges (see figure 2). Hub-nodes which are common with the top nodes based on CC are tabulated in the table 2. As it is shown in the table 3 there are 9 bottlenecks that are common with the top nodes based on stress. Action relationships between central and crucial nodes are shown in the figure 3.

## Discussion

There are several studies about colon cancer (14), gastric cancer (15) and the other types of gastrointestinal diseases that are established based on PPI network analysis. In this study determination of early stage biomarkers, identification of drug targets, and presents of new perspective of CD molecular mechanism are investigated. Since serum, cell lines, and tissue are suitable sources for sample preparation, intestinal tissue of celiac patients is selected to be analyze based on gene expression pattern. As it is shown in the figure 1 the gene expression profiles of the patients and controls are median centered, thus they are comparable statistically. It is expected that there are several differential expressed genes which can be use as discriminable tool to separate patients from controls. However, there are large numbers of DEGs but like the other study the genes cannot included a scale free network. The possible manner to construct a scale free network by adding adequate numbers of related neighbor genes is represented in the table 1. As it is depicted in the figure 2 adding the limited numbers of relevant genes to query genes was lead to scale free network. Central nodes such as hubs and bottlenecks are introduced as critical nodes of the many disease networks. In the tables 2 and 3 the hubs and bottlenecks which at least are highlighted based on other centrality parameters are identified. It must be mentioned that these 16 central nodes are selected among the query genes and not from the added neighbor genes. A powerful central node among hubs and bottlenecks is VEGFA and is the only hub-bottleneck node among the hubs and bottleneck which is included the top nodes based on closeness centrality and stress. Highlighting VEGFA as a critical DEG assumes that it is involved deeply in the regulation of the other central nodes. In the figure 3 crucial role of VEGFA as a regulatory DEG is emphasized. VEGFA binds to AOC3 and activates and up regulates LEP. Based on the findings it seems that VEGFA plays a critical role in the CD. Following discussion is designed to find interfering VEGFA with CD.

 VEGF and its receptor are both important in angiogenesis under physiological and pathological conditions, it is reported that VEGF plays crucial role in cancer promotion. VEGFA as one member of VEGF family is also involve in angiogenesis (16). It is reported that small bowel mucosal of CD patients generates immunologically active molecules which effect liver of patients. In this regard, high levels of angiogenic factors such as VEGF cause development of vascular lesions in CD patients. There is evidence that mucosal VEGA is overexpressed in CD patients (17).

Potent role of VEGF in angiogenic, mitogenic, permeability, and fibrosis enhancing peptide in collagenous colitis is reported in 2004 by Yesuf Taha et al*.*. In this study, increased perfusion of VEGF from descending colon and rectum of patients is confirmed; however, serum level of VEGF is unchanged (18). VEGF and EGFR are included in a PPI network that was constructed for celiac patents. The samples were human peripheral blood mononuclear cells. However, both EGFR and VEGF were not included in the query proteins. These tow proteins were added to the query proteins as related neighbors (7). Since VEGF expression change is reported for several diseases such as different types of cancers it is not suitable as specific biomarker or for celiac but it can be used for patients’ follow up.

## Conflict of interests

The authors declare that they have no conflict of interest.
